# *Cafestol* ameliorates diabetic nephropathy via Keap1-Nrf2 axis activation: A novel renoprotective mechanism independent of glycemic control

**DOI:** 10.1371/journal.pone.0349192

**Published:** 2026-05-14

**Authors:** Nujud A. Sinan, Mona S. Almujaydil

**Affiliations:** Department of Food Science and Human Nutrition, College of Agriculture and Food, Qassim University, Buraydah, Saudi Arabia; Guangdong Nephrotic Drug Engineering Technology Research Center, Institute of Consun Co. for Chinese Medicine in Kidney Diseases, CHINA

## Abstract

Diabetic nephropathy is an early complication of diabetes, which triggers lipotoxicity, oxidative stress, inflammation, fibrosis, and apoptosis. This study aimed to investigate the possible protective effects of *Cafestol* and the underlying molecular mechanisms in type 1 diabetes-induced nephropathy. One dose of streptozotocin (65 mg/kg, i.p.) was used to induce diabetes, then *Cafestol* was given orally at 5 and 10 mg/kg for eight weeks. In the control rats, *Cafestol* (10 mg/kg) did not cause any significant changes to any of the measured parameters, including 24-hour urine volume, urinary albumin, creatinine, and renal injury parameters (KIM-1, NGAL, nephrin), suggesting that *Cafestol* has no adverse renal effect in normal physiology. In T1DM induction rats, renal dysfunction was apparent as marked increases in urine output, enzymatic indicators of injury, inflammatory mediators (IL-6, TNF-α, ICAM-1, nuclear NF-κB), oxidative stress (MDA), and glycation markers (AGEs, sRAGEs), while there were significant decreases in antioxidant defenses (HO-1, SOD, GSH) and nuclear Nrf2 expression. Treatment with *Cafestol* at doses of 5 mg/kg and 10 mg/kg significantly reduced the pathological parameters when compared to T1DM alone in a dose-dependent manner. *Cafestol* treatment reduced glomerular and tubular injury, inhibited renal inflammation, restored oxidative balance, and improved antioxidant capacity. *Cafestol* treatment also reduced renal Keap1 mRNA expression and increased cytoplasmic and nuclear Nrf2 protein levels, indicating that *Cafestol* activates the Nrf2 pathway without altering Nrf2 mRNA expression. Further, co-administration of Brusatol, an Nrf2 inhibitor, eliminated the renal protection effects of *Cafestol* for all measured parameters and returned levels to T1DM alone. The results suggest that *Cafestol* provides substantial renal protective, anti-inflammatory, and antioxidative benefits in a model of diabetes, mainly through the activation of Nrf2. This finding suggests that *Cafestol* may be a novel agent to activate Nrf2, which may have implications for the treatment of DN.

## Introduction

Diabetic nephropathy, or diabetic kidney disease, is a significant microvascular consequence of diabetes mellitus (DM) [1]. Diabetic nephropathy (DN) is a primary contributor to end-stage renal disease (ESRD) worldwide, impacting roughly 30–40% of individuals with diabetes [1,2]. Chronic hyperglycemia and dyslipidemia initiate the development of diabetic nephropathy by inducing oxidative stress, inflammation, fibrosis, and apoptosis, ultimately affecting the glomeruli and tubules [3,4]. Oxidative stress, resulting from elevated production of reactive oxygen species (ROS), diminishes and enhances the antioxidant mechanism, serving as the crucial initiator of all the pathogenic processes [5,6]. Consequently, managing hyperglycemia is the foremost objective in the therapy of diabetic nephropathy. To enhance pathophysiology, oral hypoglycemic agents employed in diabetes management must augment both insulin secretion and sensitivity while diminishing hepatic glucose production through the downregulation of gluconeogenesis [7]. However, there are several risks associated with synthetic oral hypoglycemic agents, and studies have shown that DN-relieving oral hypoglycemic therapies lead to obesity [8].

It has been indicated that nuclear factor erythroid 2-related factor 2 (Nrf2) inhibition has also been reported to be an important mechanism of increased oxidative stress in diabetic nephropathy. Besides, Nrf2 is a potent anti-inflammatory, fibrosis, cell death, and lipogenesis inhibitor through the activation of many transcription factors and signaling proteins [6,9,10]. Activation of Nrf2 has, therefore, been shown to improve kidney function and structure in diabetic kidney lipidosis [11–14].

Although there is partial renoprotection by current therapies, such as renin-angiotensin system inhibitors and sodium-glucose cotransporter-2 (SGLT2) inhibitors, of greater importance is the need for novel drugs with multiple disease target actions [15,16]. In this regard, natural products exhibiting anti-inflammatory and antioxidant activities have become more prominent. Recent studies have further emphasised their therapeutic potential in diabetic nephropathy by modulating inflammatory pathways and complex molecular mechanisms, highlighting their potential as therapeutic agents [17,18]. Regular coffee consumption has been consistently associated with a reduced risk for a variety of metabolic and cardiovascular diseases, including diabetes mellitus, obesity, insulin resistance, dyslipidemia, and cardiovascular disease [19–21]. Furthermore, epidemiological evidence shows that consumption of coffee is related to a reduced risk of kidney disease. These health benefits are largely attributed to coffee’s high content of bioactive compounds, particularly chlorogenic acid and the diterpenes *cafestol* and kahweol, which exhibit potent antioxidants and anti-inflammatory properties [22,23]. *Cafestol*, a diterpene and lipid-soluble ester found in green coffee beans, has been identified as a bioactive compound with multiple pharmacological properties [22]. Evidence from both in vivo and in vitro studies has demonstrated its capacity to enhance insulin secretion and promote glucose uptake [24,25]. Furthermore, *cafestol* exhibits significant anti-inflammatory effects, primarily through the inhibition of pro-inflammatory mediators such as NADPH oxidase 2 (Nox2), inducible nitric oxide synthase (iNOS), and the downregulation of the nuclear factor-kappa B (NF-κB) signaling pathway [26,27]. Notably, *cafestol* has also been found to activate the Nrf2 signalling pathway, which plays a crucial role in cellular defence against oxidative damage in diabetic cardiomyopathy and nephropathy [23,28,29].

Diabetic nephropathy is a progressive renal disorder resulting from persistent hyperglycemia and oxidative stress, which induces inflammation, fibrosis, and renal impairment. Existing therapies are constrained, and *cafestol*, a bioactive compound found in coffee, is postulated to safeguard the kidneys by diminishing oxidative stress and inflammation, thereby enhancing renal function in diabetic rats. The study aims to assess the preventive effects of *cafestol* on diabetic nephropathy utilizing a streptozotocin-induced diabetic rat model. The study investigates the effects of *cafestol* on renal function, histological changes in the kidneys, oxidative stress markers, and inflammatory responses, aiming to clarify its potential as a therapeutic agent for the management of diabetic nephropathy.

## Materials and methods

### Animal

Adult male Wistar rats (weighing 180 ± 15 g and approximately 8 weeks old) were used and housed at the Department of Food Science and Human Nutrition, College of Agriculture and Food, Qassim University, Saudi Arabia, in standard laboratory cages with appropriate bedding and maintained under controlled conditions, ensuring a stable temperature (20–22°C) and humidity levels (40–60%). To provide a consistent environment and minimise external influences, a 12-hour light/dark cycle was maintained. The rats had unrestricted access to standard food and water throughout the acclimatisation and experimental phases. All animal handling and experimental protocols were conducted in accordance with ethical guidelines. They were approved by the Committee of Research Ethics of Qassim University, Saudi Arabia (approval no. 24-14-01/2024) in compliance with International Animal Ethics, and in accordance with the ARRIVE Essential 10 criteria.

### Induction of the T1DM model

T1DM was induced in rats via a single intraperitoneal injection of streptozotocin (STZ, 65 mg/kg; Sigma Aldrich, 142155) freshly dissolved in 0.5 M sodium citrate, as established by others [30,31]. To prevent hypoglycemia, animals received a 0.5% oral glucose solution for three days post-injection. On day 7, blood glucose levels were measured from tail vein samples after anesthesia with a ketamine/xylazine hydrochloride mixture (80/10 mg/kg). Rats with glucose levels >320 mg/dL were classified as diabetic and included in subsequent experiments.

### Experimental design

Cafestol acetate (C₂₂H₃₀O₄) powder (purity > 98%) (#81760-48-7) was purchased from Sigma and freshly dissolved in 0.5% carboxymethylcellulose (CMC). This study included five animal groups (n = 12 rats/group). The Control group consisted of non-diabetic rats receiving only 0.5% CMC orally as a vehicle. The Control + *Cafestol*-treated group included non-diabetic rats administered *Cafestol* solution (10 mg/kg) orally. The T1DM-model group comprised diabetic rats treated with an oral dose of 0.5% CMC. The T1DM + *Cafestol*-treated groups included diabetic rats receiving oral *Cafestol* solution at low and high doses (5 and 10 mg/kg). Lastly, the T1DM + *Cafestol* + Brusatol-treated group consisted of diabetic rats treated with *Cafestol* (10 mg/kg) along with an intraperitoneal dose of Brusatol, an Nrf2 inhibitor (2 mg/kg). All treatments were administered thrice per week. Brusatol was always given 1 hour before administering *Cafestol*. Experiments continued for 8 weeks; a duration established as necessary for developing diabetic nephropathy after such STZ dose administration [32].

### Dose selection

Brusatol was used according to established guidelines from earlier studies (to lower the in vivo Nrf2 nuclear translocation and activity across several tissues, including the heart, intestines, and kidneys [30,33]. Furthermore, our early preliminary results showed that oral *Cafestol* therapy caused a dose-dependent drop in urinary albumin at doses ranging from 1 to 20 mg/kg, with most effects seen at 10, 15, and 20 mg/kg. Previously, a 5 mg/kg dose of *Cafestol* was demonstrated to protect rats from doxorubicin-induced heart damage [23]. *Cafestol* treatment at 7 and 14 mg/kg also produced roughly hypoglycemic effects in non-diabetic obesity patients [19].

### Urine and serum collection

Twenty-four hours after the final treatment, rats were placed in metabolic cages for a 2-hour urine collection. Following a 12-hour fasting period, they were anesthetized with a ketamine/xylazine hydrochloride mixture (80/10 mg/kg). Blood samples were then obtained via cardiac puncture and collected into either gel-containing or EDTA-coated tubes. The samples were left at room temperature for 30 minutes before being centrifuged at 500 × g for 15 minutes to separate the supernatant, yielding serum and plasma. All plasma, serum, and filtered urine samples were immediately stored at −20°C for future analysis. All urine, serum, and plasma samples were coded before the biochemical evaluation, and all analyses were performed by investigators blinded to treatment group allocation until the analyses were completed

### Preparation of total kidney homogenates

After humanely euthanizing the rats using a two-step method in accordance with the consulting American Veterinary Medical Association (AVMA) Guidelines for the Euthanasia of Animals, 2020: first, by inhalation of isoflurane, second, after the animal is unconscious, physical cervical dislocation was done, to confirm death definitively, both kidneys were aseptically removed, cleaned of excess blood, weighed individually, and immediately frozen at −80°C for long-term storage. Detailed records were kept, including kidney weight, animal ID, and collection time. To prepare the total kidney extracts, 30 mg of frozen kidney tissue from each sample was carefully taken and kept on ice to maintain protein stability. The kidney tissue was first washed in cold phosphate-buffered saline (PBS) to remove residual blood. The tissue was then homogenized in a buffer containing 50 mM Tris-HCl (pH 7.4), 150 mM NaCl, 1 mM EDTA, and 0.1% Triton X-100, with a protease inhibitor cocktail. The homogenized sample was centrifuged at 10,000 × g for 15 minutes at 4°C to remove cellular debris. The supernatants obtained from all samples, which contained soluble proteins, were collected carefully, labelled, and stored at −80°C for further biochemical analysis.

### Preparation of nuclear kidney extract

Nuclear protein was isolated from 30 mg of frozen kidney tissue by homogenization in 500 µL of ice-cold PBS with inhibitors, followed by lysis with hypotonic buffer containing NP-40. After centrifugation at 1,000 × g for 5 minutes at 4°C, the cytoplasmic supernatant was stored, and the nuclear pellet was further processed in a detergent-free buffer. The final nuclear fraction was obtained by centrifugation at 14,000 × g for 10 minutes at 4°C.

### Biochemical analysis in plasma and serum

Insulin levels were measured in plasma using the Ann Arbor ELISA Kit (589501, TX, USA), while glucose concentrations in the same samples were assessed with the Cayman Chemicals ELISA Kit (10009582, CA, USA). Plasma Hemoglobin A1c (HbA1c) levels were determined using the BioTrend Chemicals HbA1c ELISA Kit (# CC-80300, BioTrend Chemicals, Köln, Germany), and serum soluble receptor for advanced glycation end products (sRAGE) was evaluated with the MyBioSource Rat sRAGE ELISA Kit (# MBS7606870, MyBioSource, CA, USA). Serum levels of total cholesterol, triglycerides, high-density lipoprotein cholesterol (HDL-c), and low-density lipoprotein cholesterol (LDL-c) were measured using assay kits provided by AFG Scientific, IL, USA (EK720559, EK720636, EK720660) and # EK720763, respectively. All measurements were conducted in duplicate for 12 serum samples per group, adhering to the manufacturer’s protocols.

### Biochemical analysis in the urine

The levels of albumin and creatinine in the urine were measured using assay kits (80662, AFG Scientific, IL, USA, and # 80340, Crystal Chem). Markers of glomerular and tubular function, including kidney injury molecule-1 (KIM-1), Neutrophil gelatinase-associated lipocalin (NGAL), and nephrin, were assessed using various ELISA kits (# EK720751; AFG Scientific, IL, USA; # 80687, Crystal Chem, CA, USA; and CSB-E13957r, CusaBio, MI, USA, respectively). All measurements were performed in duplicate for 12 urine samples per group, following the manufacturer’s protocols.

### Biochemical measurement in the kidney total and nuclear homogenate

The levels of TNF-α were quantified using the ThermoFisher ELISA Kit (# BMS622, Frankfurt, Germany). The levels of Intracellular cell-adhesion molecule-1 (ICAM-1) were measured with the Abcam ELISA Kit (Catalog #ab100763, Cambridge, UK). The levels of TNF-α were quantified using the ThermoFisher ELISA Kit (# BMS622, Frankfurt, Germany), Malondialdehyde (MDA), superoxide dismutase (SOD), heme oxygenase-1 (HO-1), and glutathione reductase (GSH) levels were quantified using ELISA kits from AFG Scientific (Catalog #EK720188, #EK720889, #EK720658, and #EK720424, respectively, IL, USA). NF-κB protein levels in the nuclear extract were measured using the MyBioSource ELISA Kit (Catalog #MBS752046, CA, USA). The activity of Nrf2 in the nuclear extracts was assessed using the Abcam rat-specific ELISA Kit (Catalog #ab2072223, Cambridge, UK), and the levels of Kelch-like ECH-associated protein 1 (Keap1) were quantified with an ELISA kit from MyBioSource (Catalog #MBS7218529m, CA, USA). Interleukin-6 (IL-6) levels were quantified with a kit from Lab Science (Catalog #E-EL-R0015, TX, USA). Each experimental group consisted of 12 samples, and all procedures were performed strictly according to the protocols provided by the respective kit manufacturers.

### Real-time polymerase chain reaction (qPCR)

To assess the mRNA expression of Keap1 and Nrf2 in kidney tissues, we utilized a quantitative Real-Time PCR (qPCR) approach. RNA was extracted from frozen kidney samples with the RNeasy Mini Kit (Catalog #74004, Qiagen, Hilden, Germany), and its integrity and concentration were evaluated using a Nanodrop spectrophotometer, which measured absorbance ratios at 260/280 nm. cDNA synthesis was carried out using the Thermo Fisher cDNA Synthesis Kit (Catalog #K1621, Thermo Fisher, Waltham, MA, USA). The following primers were designed for the amplification of the target genes: Keap1 was amplified with the forward primer 5′-CTTCGGGGAGGAGGAGTTCT-3′ and reverse primer 5′-CGTTCAGATCATCGCGGCTG-3′, producing a 132 base pair product (Accession #NM_057152.2). For Nrf2, the forward primer was 5′-AAAATCATTAACCTCCCTGTTGAT-3′, and the reverse primer was 5′-CGGCGACTTTATTCTTACCTCTC-3′, with an amplicon size of 118 base pairs (Accession #NM_031789). β-actin was used as a reference gene with the forward primer 5′-GACCTCTATGCCAACACAGT-3′ and reverse primer 5′-CACCAATCCACACAGAGTAC-3′, generating a 154 base pair product (Accession #NM_031144). The qPCR reactions were carried out on a Bio-Rad system using the Ssofast EvaGreen Supermix (Catalog #172–5200, Bio-Rad, Hercules, CA, USA). Each 20 µL reaction included 10 µL of EvaGreen master mix, 0.2 µL of each primer (final concentration of 500 nM), 2 µL of cDNA template (50 ng final concentration), and 7.6 µL of nuclease-free water. The amplification protocol began with an initial denaturation step at 98°C for 30 seconds, followed by 40 cycles at 98°C for 5 seconds and 60°C for 5 seconds. A melting curve analysis was performed to verify amplification specificity. mRNA expression levels of Keap1 and Nrf2 were normalized to β-actin using the ΔΔCt method. Data were analyzed using the qPCR system software, strictly following the manufacturer’s protocols.

### Histological study

Kidney tissues were fixed in formalin, dehydrated through graded ethanol, embedded in paraffin, and sectioned at 5–7 µm. Sections were mounted on slides, deparaffinized with xylene, and rehydrated using a reverse ethanol gradient. Slides were stained with hematoxylin for 5–10 minutes and then eosin for 1–3 minutes to stain the cytoplasm and extracellular matrix, dehydrated through graded ethanol, cleared with xylene, and mounted. Histological analysis was performed using an Olympus CX23 light microscope (Olympus Corporation, Tokyo, Japan). Histological sections were coded before microscopic evaluation, and histological analysis was performed by investigators blinded to treatment group allocation until the analyses were completed.

### Statistical analysis

GraphPad Prism software version 8 performed statistical analyses of all data. Before statistical analysis, the normality of the data was assessed using the Kolmogorov-Smirnov test. Analysis was done by two-way ANOVA, and Tukey’s post hoc test was used to determine a significant difference with a p-value less than 0.05. For all datasets, data are expressed as mean ± SD.

## Results

### Comparative effects of *Cafestol* and Brusatol on metabolic and lipid parameters

According to Table 1, Control + *Cafestol* (10 mg/kg) showed no significant changes in body weight, food intake, and plasma glucose, insulin, or HbA1c levels, nor serum levels of total TGs, total CHOL, LDL-c, FFAs, and HDL-c compared to the control group. Conversely, T1DM rats showed significantly lower body weight, higher food intake, and increased plasma glucose, insulin, and HbA1c compared to control rats. Additionally, T1DM rats had significantly elevated serum total TGs, CHOL, LDL-c, and FFAs and showed significantly lower HDL-c than control rats. However, no significant changes in the levels of these parameters were observed when comparing T1DM + *Cafestol* (5 mg/kg) and T1DM + *Cafestol* (10 mg/kg) with each other, or with T1DM + *Cafestol* (10 mg/kg) + Brusatol (2 mg/kg). Moreover, no significant differences were depicted in body weights, food intake, plasma glucose, insulin, HbA1c, and lipid profile when all treated groups were compared with the T1DM model rats.

**Table 1:** Changes in food intake and final body weights, as well as alterations in some diabetic indicators and lipid profiles in all experimental groups.

	Traits	Control	Control + *Cafestol* (10 mg/kg)	T1DM	T1DM + *Cafestol* (5 mg/kg)	T1DM + *Cafestol* (10 mg/kg)	T1DM + *Cafestol* (10 mg/kg) + Brusatol (2 mg/kg)
	FBW	516 ± 41.3	527 ± 55.6	347 ± 28.6^ab^	351.5 ± 36.5^ab^	342 ± 30.6^ab^	355.5 ± 29.5^ab^
	WFI(g)	256 ± 23.2	248 ± 20.9	364 ± 31.6^ab^	351 ± 38.7^ab^	349 ± 32.7^ab^	355 ± 33.8^ab^
Plasma	Glucose (mg/dl)	96.5 ± 7.8	99.8 ± 10.5	343.2 ± 29.7^ab^	321.5 ± 34.3^ab^	339.5 ± 44.1^ab^	344.4 ± 39.3^ab^
	Insulin (ng/ml)	4.3 ± 0.39	4.1 ± 0.56	8.6 ± 0.91^ab^	7.9 ± 0.87^ab^	8.1 ± 0.83^ab^	8.8 ± 0.93^ab^
	HbA1c (%)	5.3 ± 0.58	5.0 ± 0.63	7.6 ± 0.64^ab^	7.9 ± 0.74^ab^	7.1 ± 0.88^ab^	7.8 ± 0.81^ab^
Serum	TGs (mg/dL)	81.4 ± 9.7	88.5 ± 7.6	234.5 ± 25.6^ab^	236.9 ± 23.4^ab^	258.4 ± 18.7^ab^	243.4 ± 29.4^ab^
	CHOL (mg/dL)	95.4 ± 10.5	87.8 ± 9.2	256.7 ± 27.5^ab^	273.4 ± 30.1^ab^	267.8 ± 29.4^ab^	249.5 ± 28.9^ab^
	LDL-c (mg/dL)	44.7 ± 5.8	39.7 ± 4.1	124.5 ± 14.5^ab^	119.5 ± 10.8^ab^	128.4 ± 11.7^ab^	119.8 ± 13.4^ab^
	HDL-c (mg/dl)	31.3 ± 3.9	37.6 ± 4.6	15.7 ± 1.4^ab^	16.6 ± 1.8^ab^	15.1 ± 1.9^ab^	16.8 ± 2.1^ab^

Data are presented as mean ± SD (n = 8 rats per group). Statistical analysis was performed using two-way ANOVA followed by Tukey’s post hoc test. Statistical significance was accepted at p < 0.05. a significantly different from the Control group; b significantly different from the Control + Cafestol (10 mg/kg) group; c significantly different from the T1DM group; d significantly different from the T1DM + Cafestol (5 mg/kg) group; e significantly different from the T1DM + Cafestol (10 mg/kg) group. Brusatol: selective Nrf2 inhibitor. FBW: final body weight; WFI: weekly food intake.

### *Cafestol* enhances urine output and ameliorates kidney injury markers in T1DM rats in a dose-dependent and Nrf2 Manner

**Fig 1:**
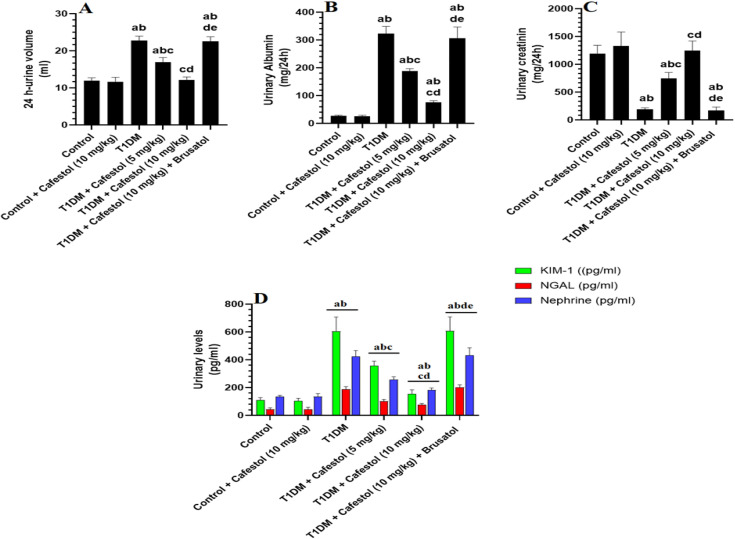
Effects of *Cafestol* on renal function and injury markers in control and diabetic rats. Data are presented as mean ± SD (n = 8 rats per group). Statistical analysis was performed using two-way ANOVA followed by Tukey’s post hoc test. Statistical significance was accepted at p < 0.05. a significantly different from the Control group; b significantly different from the Control + *Cafestol* (10 mg/kg) group; c significantly different from the T1DM group; d significantly different from the T1DM + *Cafestol* (5 mg/kg) group; e significantly different from the T1DM + *Cafestol* (10 mg/kg) group. Brusatol: selective Nrf2 inhibitor.

Fig 1 indicates that compared to the control group, administration of *Cafestol* (10 mg/kg) alone does not induce significant alterations in 24-hour urine volume, urinary albumin levels, or urinary creatinine levels, as well as in urinary markers of glomerular and tubular injury (KIM-1, NGAL, and nephrin), suggesting that *Cafestol* has no adverse effect on renal function in healthy rats. In contrast, T1DM induction led to a substantial increase in 24-hour urine volume, urinary albumin levels, and urinary creatinine levels, along with a pronounced and significant elevation in urinary levels of KIM-1, NGAL, and nephrin, indicating severe glomerular and tubular damage.

Fig 1 also shows that treatment group with *Cafestol* at both 5 mg/kg and 10 mg/kg doses in T1DM rats significantly mitigated these pathological changes, as evidenced by a dose-dependent reduction in urine output and urinary levels of albumin, creatinine, KIM-1, NGAL, and nephrin levels, suggesting an overall renoprotective effect in diabetic conditions with the highest dose of *Cafestol*. However, when Brusatol, an Nrf2 inhibitor, was co-administered with Cafestol (10 mg/kg), the beneficial effects of *Cafestol* were largely reversed. Specifically, a combination of Brusatol with Cafestol led to a significant increase in 24-hour urine volume, urinary albumin, and urinary creatinine, along with an elevation in urinary KIM-1, NGAL, and nephrin levels. Notably, the 24-hour urine output and the urinary levels of all the above markers were not significantly different when T1DM rats were compared with T1DM + *Cafestol* (10 mg/kg) + Brusatol-treated rats

### *Cafestol* Attenuates renal inflammation in T1DM Rats in a dose-dependent manner through Nrf2 activation

**Fig 2:**
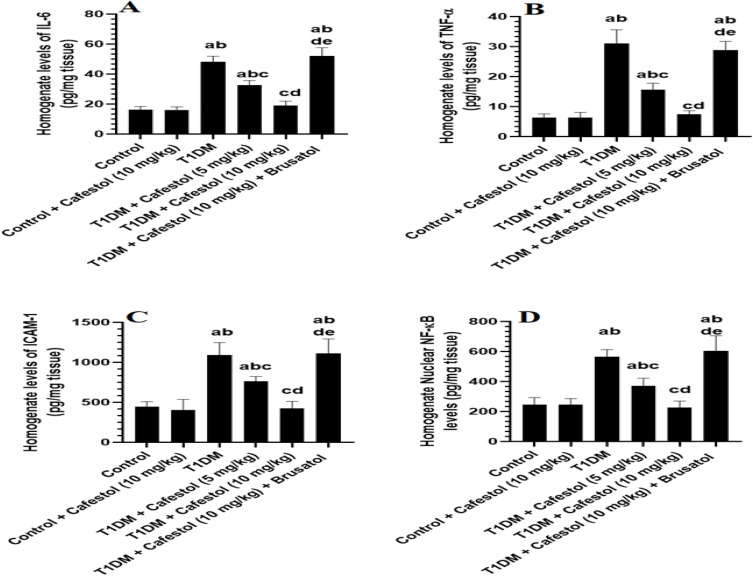
Effects of *Cafestol* on renal inflammatory markers in control and diabetic rats. Data are presented as mean ± SD (n = 8 rats per group). Statistical analysis was performed using two-way ANOVA followed by Tukey’s post hoc test. Statistical significance was accepted at p < 0.05. a significantly different from the Control group; b significantly different from the Control + *Cafestol* (10 mg/kg) group; c significantly different from the T1DM group; d significantly different from the T1DM + *Cafestol* (5 mg/kg) group; e significantly different from the T1DM + *Cafestol* (10 mg/kg) group. Brusatol: selective Nrf2 inhibitor.

Fig 2 shows no significant alteration in renal total homogenate levels of major inflammatory markers, including IL-6, TNF-α, and ICAM-1, nor in the nuclear NF-κB levels between Control and *Cafestol* (10 mg/kg)-treated rats. The renal homogenates of T1DM rats demonstrated a significant inflammatory response, marked by a pronounced upregulation of IL-6, TNF-α, and ICAM-1, along with increased nuclear NF-κB activation in comparison to Control rats. This indicates a heightened state of renal inflammation and an intensified activation of pro-inflammatory signaling pathways. Compared with T1DM rats, *Cafestol* treatment at 5 mg/kg and 10 mg/kg significantly mitigated these inflammatory changes, as evidenced by a dose-dependent reduction in renal levels of IL-6, TNF-α, ICAM-1, and nuclear NF-κB (Fig 2A-D). The data suggests that *Cafestol* exerts an anti-inflammatory effect, possibly through modulation of NF-κB signaling. However, the co-administration of Brusatol, an Nrf2 inhibitor, with *Cafestol* (10 mg/kg) in T1DM rats reversed these protective effects as compared to T1DM + *Cafestol* (5 and 10 mg/kg), where there were significant increases in the renal levels of IL-6, TNF-α, ICAM-1, and nuclear NF-κB levels (Fig 2A-D). In addition, the renal levels of all these inflammatory markers showed no significant changes when T1DM rats were compared with T1DM + *Cafestol* (10 mg/kg) + Brusatol rats.

### *Cafestol* attenuates T1DM-induced renal oxidative stress and glycation markers via Nrf2 activation in a dose-dependent manner

**Fig 3:**
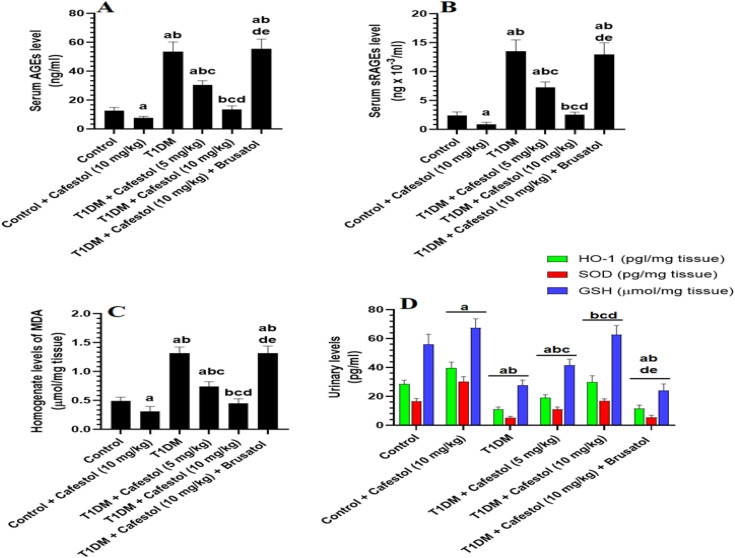
Effects of *Cafestol* on renal oxidative stress/antioxidant markers in control and diabetic rats. Data are presented as mean ± SD (n = 8 rats per group). Statistical analysis was performed using two-way ANOVA followed by Tukey’s post hoc test. Statistical significance was accepted at p < 0.05. a significantly different from the Control group; b significantly different from the Control + *Cafestol* (10 mg/kg) group; c significantly different from the T1DM group; d significantly different from the T1DM + *Cafestol* (5 mg/kg) group; e significantly different from the T1DM + *Cafestol* (10 mg/kg) group. Brusatol: selective Nrf2 inhibitor.

The administration of *Cafestol* (10 mg/kg) to control rats significantly lowered levels of advanced glycation end products (AGEs) and soluble receptor for advanced glycation end products (sRAGEs), as well as renal homogenate levels of malondialdehyde (MDA), as compared to Control rats, as shown in Fig 3. It also significantly increased renal homogenate levels of GSH, HO-1, and SOD as compared to control rats (Fig 3A-D). However, in Type 1 diabetes mellitus (T1DM) rats, a profound increase in serum levels of AGEs and sRAGEs, alongside renal levels of MDA, was observed, accompanied by a marked reduction in HO-1, SOD, and GSH levels as compared to Control rats, reinforcing the notion of severe oxidative imbalance and compromised antioxidant defenses in diabetic conditions. When compared to T1DM rats, the administration of *Cafestol* at either 5 mg/kg or 10 mg/kg to T1DM rats significantly ameliorated these pathological alterations, as evidenced by the significant decline in serum levels of AGEs and sRAGEs, reduction in renal MDA levels, and the substantial restoration of HO-1, SOD, and GSH levels (Fig 3). However, these effects were dose-dependent and were more profound on all parameters with the 10 mg/kg dose of *Cafestol*.

These findings underscore potent antioxidative and glycation-inhibitory properties of *Cafestol* in a dose-dependent manner, highlighting its therapeutic potential in mitigating diabetes-induced oxidative stress and related complications. Importantly, co-administration of Brusatol along with *Cafestol* in T1DM rats completely abrogated the protective effects of this drug, as reflected by the re-elevation of serum levels of AGEs, sRAGEs, and renal MDA levels that were coupled with the significant depletion of HO-1, SOD, and GSH as compared to T1DM rats that were treated with the 5 or 10 mg/kg *Cafestol*.

Fig 3 also shows that no significant changes were observed in the serum and renal levels of all these markers when T1DM + *Cafestol* (10 mg/kg) + Brusatol rats were compared to T1DM rats. This reversal strongly indicates that the beneficial effects of *Cafestol* on oxidative stress and glycation markers are mechanistically dependent on Nrf2 activation.

### *Cafestol* activates renal Nrf2 cytoplasmic and nuclear translocation by downregulating Keap1 in both control and T1DM rats

**Fig 4:**
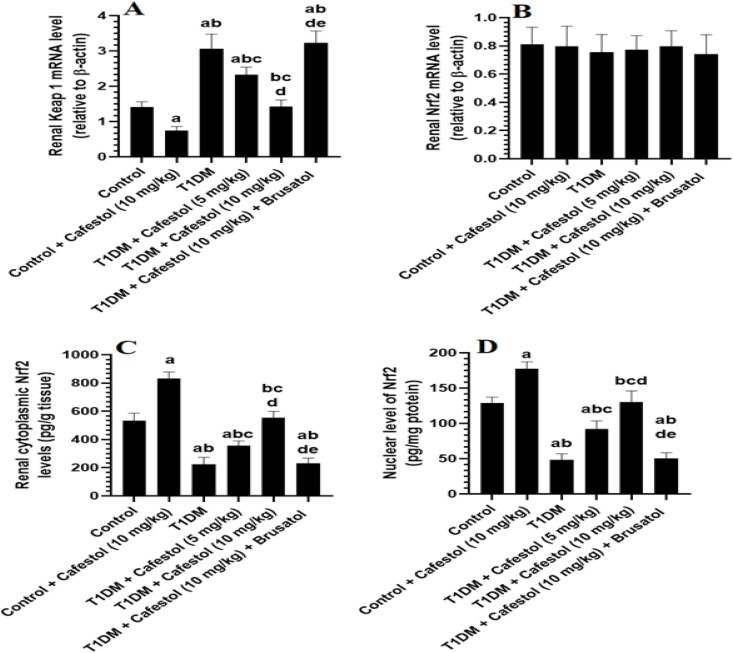
Effects of *Cafestol* on renal Keap1/Nrf2 pathways in control and diabetic rats. Data are presented as mean ± SD (n = 8 rats per group). Statistical analysis was performed using two-way ANOVA followed by Tukey’s post hoc test. Statistical significance was accepted at p < 0.05. a significantly different from the Control group; b significantly different from the Control + *Cafestol* (10 mg/kg) group; c significantly different from the T1DM group; d significantly different from the T1DM + *Cafestol* (5 mg/kg) group; e significantly different from the T1DM + *Cafestol* (10 mg/kg) group. Brusatol: selective Nrf2 inhibitor.

Fig 4 displays *Cafestol* Activates Renal Nrf2 Cytoplasmic and Nuclear Translocation by downregulating Keap1 in Both Control and T1DM Rats. In control rats, administration of *Cafestol* (10 mg/kg) did not significantly alter renal mRNA levels of Nrf2 but significantly reduced Keap1 mRNA expression, which was accompanied by marked increases in both cytoplasmic and nuclear Nrf2 protein levels, indicating that *Cafestol* activates basal Nrf2 signaling under physiological conditions. In contrast, T1DM rats exhibited a substantial upregulation of Keap1 mRNA expression compared to control rats, accompanied by a significant reduction in renal cytoplasmic and nuclear Nrf2 protein levels, despite no apparent changes in Nrf2 mRNA expression. The administration of *Cafestol* at either 5 mg/kg or 10 mg/kg to T1DM rats significantly mitigated these alterations, as evidenced by a marked reduction in Keap1 mRNA expression and a concomitant increase in nuclear Nrf2 protein levels, despite no significant changes in Nrf2 mRNA expression (Fig 4). This restoration of Nrf2 signaling by *Cafestol* was dose-dependent, with the 10 mg/kg dose exerting a more pronounced effect. However, co-administration of Brusatol, an Nrf2 inhibitor, with *Cafestol* (10 mg/kg) in T1DM rats abolished the *Cafestol*-induced reduction in Keap1 mRNA expression and the increments in the cytoplasmic and nuclear Nrf2 protein levels (Fig 4A-D). This reversal strongly indicates that the protective effects of *Cafestol* on Nrf2 signalling are mediated through an Nrf2-dependent mechanism, further supporting its role in enhancing Nrf2 nuclear translocation and activity in the diabetic kidney.

## Discussion

The findings from the streptozotocin-induced T1DM rat model elucidate the characteristic pathophysiological features of untreated T1DM, including cachexia, increased food consumption, hyperglycemia, hyperinsulinemia, elevated HbA1c levels, and dyslipidemia. *Cafestol* supplementation at 10 mg/kg did not significantly alter these parameters in non-diabetic controls, indicating its neutral effect in healthy states. In T1DM models, *cafestol* (5 or 10 mg/kg) and its combination with brusatol failed to improve body weight, food intake, glycemia, or lipid profiles, suggesting limitations related to dosage, duration, or model-specific factors affecting beta-cell function [34,35]. While *cafestol* shows efficacy in type 2 diabetes models by lowering glucose and improving insulin sensitivity, its effects in T1DM appear limited, primarily benefiting insulin-resistant states rather than addressing severe hyperglycemia or oxidative stress pathways [36].

Diabetes mellitus induces hyperglycemia, which can progress to micro- and macrovascular problems and ultimately become a primary cause of death. Since increased ATP dephosphorylation following streptozotocin administration provides a substrate for xanthine oxidase, which in turn produces superoxide radicals, hydrogen peroxide, and hydroxyl radicals, the mechanism of STZ as a diabetogenic agent is mediated by reactive oxygen species [37]. Hyperglycemia-driven oxidative stress underlies much of the diabetic pathology observed here. STZ’s diabetogenic effect involves ATP depletion and excessive ROS production via xanthine oxidase activation [38,39]. Our T1DM group exhibited hallmark metabolic derangements and dyslipidemia consistent with prior reports, including elevated triglycerides, cholesterol, LDL, and free fatty acids alongside reduced HDL [40–42].

Results revealed that the control group’s metabolic parameters were not significantly altered by *cafestol* (10 mg/kg), indicating that *cafestol* at this dosage does not significantly disrupt normal physiological or metabolic homeostasis [23]. A comparison of T1DM + *Cafestol* (10 mg/kg) with T1DM + Cafestol (5 mg/kg) did not show any significant difference in the metabolic and lipid parameters. Similarly, in groups of T1DM + Cafestol (10 mg/kg) + Brusatol (2 mg/kg). All the *cafestol*-treated groups, when compared with untreated T1DM models, did not display significant differences in terms of body weight, food intake, plasma glucose, insulin, HbA1c, and lipid parameters. The findings suggest that neither *cafestol* alone nor *cafestol* together with brusatol significantly ameliorated the metabolic and lipid abnormalities associated with T1DM at the dosages tested in the study. It has been observed that the beneficial impact of certain compounds on metabolic parameters using the STZ model [43,44]. This requires a further investigation of the specific mechanism and dose of *cafestol* in the STZ-induced diabetes model.

The results show that *Cafestol*, at 10 mg/kg, does not adversely affect renal function in healthy rats, as indicated by stable 24-hour urine volume, urinary albumin, creatinine, and markers of glomerular and tubular injury (KIM-1, NGAL, nephrin). However, in T1DM rats, *Cafestol* treatment at 5 and 10 mg/kg reduced the pathological increases in these markers and urine output, demonstrating a dose-dependent renoprotective effect. This protective action is likely mediated through activation of the Nrf2 pathway, a key regulator of antioxidant defense that mitigates oxidative stress and inflammation in diabetic nephropathy [45]. Co-administration of Brusatol, an Nrf2 inhibitor, reversed the protective effects of *Cafestol*, leading to kidney injury markers and urine output like untreated diabetic rats, illustrating the importance of Nrf2 activation in *Cafestol’s* renoprotection. Modern studies support that *Cafestol* activates Nrf2, which in diabetic nephropathy reduces oxidative damage and fibrosis, consistent with the observed functional and biochemical improvements [46,47]. On the other hand, blocking Nrf2 makes kidney damage worse in diabetes by letting oxidative stress and fibrotic signaling go unchecked, as shown by Brusatol co-treatment [48].

Findings showed that in the control group, *cafestol* (10 mg/kg) alone could not induce significant changes in 24-hour urine volume, urinary albumin, and urinary concentrations of creatinine. Similarly, urinary glomerular and tubular injury markers include KIM-1, NGAL, and nephrin. The evidence proved that *cafestol* at this level does not harm the renal function of healthy rats [23]. T1DM, on the other hand, caused significant renal impairment, resulting in an increase in 24-hour urine volume, urinary albumin, and urinary creatinine. Urinary KIM-1, NGAL, and nephrin levels were also markedly increased, showing generalised glomerular and tubular damage and conforming to diabetic nephropathy models [49,50]. There has been considerable use of the biomarkers for early detection of diabetic kidney injury [46]. These pathological changes were reduced in T1DM treated with *cafestol* at doses of 5 mg/kg and 10 mg/kg in a dose-dependent manner. This nephroprotection was demonstrated by decreasing urine output and urinary excretion of albumin, creatinine, KIM-1, NGAL, and nephrin; the 10 mg/kg dose produced the greatest benefit, which implies that *cafestol* has therapeutic potential for preventing DN.

The findings indicate that *Cafestol* markedly alleviates renal inflammation in T1DM rats by dose-dependently decreasing critical pro-inflammatory markers IL-6, TNF-α, ICAM-1, and nuclear NF-κB levels, underscoring its anti-inflammatory function in diabetic nephropathy. These findings correspond with studies indicating that *Cafestol* suppresses pro-inflammatory cytokines and endothelial adhesion molecules by modulating NF-κB signaling, a pivotal mechanism in the inflammation and progression of diabetic kidney disease [34,51]. The simultaneous administration of Brusatol, an Nrf2 inhibitor, negates the protective benefits of *Cafestol*, highlighting the role of Nrf2 signaling in this anti-inflammatory process [51]. Nrf2 activation is recognized for its ability to inhibit NF-κB-mediated inflammatory responses by augmenting antioxidant defenses and diminishing oxidative stress, both of which are pivotal in diabetes-induced kidney inflammation [52,53]. *Cafestol’s* renal protective effects in diabetes seem to function through a crosstalk mechanism that involves Nrf2-mediated suppression of NF-κB signaling pathways, aligning with the current comprehension of diabetic nephropathy pathophysiology and the anti-inflammatory capabilities of Nrf2 activators.

*Cafestol’s* renoprotective effects rely on the Nrf2 pathway; brusatol inhibits Nrf2, blocking *cafestol’s* benefits and increasing kidney injury markers in diabetic rats. This finding has profound implications that *cafestol* does work to exert its renoprotective action, at least partially, by inducing the activation of the Nrf2 signaling pathway that is of vital importance in antioxidant defense and against oxidative stress and inflammation of DN [22,49,54]. Our findings elucidate the vast anti-inflammatory potential of *Cafestol* in T1DM-induced kidney inflammation, in addition to Nrf2 signaling causality in regulating such protective effects. To begin with, we observed that *cafestol* (10 mg/kg) administered to healthy rats did not initiate any measurable changes in the renal total homogenate level of significant inflammatory markers. This means that the tested dose of *cafestol* does not induce an inflammatory response in healthy renal tissue, and this also agrees with previous experiments demonstrating its safety and lack of pro-inflammatory effects in non-diseased states [29]. However, in renal homogenates, T1DM rats displayed an inflammatory response in contrast to controls, with markedly elevated nuclear NF-κB activation and levels of IL-6, TNF-α, and ICAM-1 expression. This is in line with the established pathophysiology of DN and implies increased renal inflammation and activation of the pro-inflammatory signalling cascade [55,56]. By encouraging cellular dysfunction and fibrosis, elevated levels of these inflammatory markers, specifically TNF-α and IL-6, are known to result in diabetic kidney injury [57,58].

Interestingly, both 5 mg/kg and 10 mg/kg of *cafestol* were effective in halting these inflammatory changes in the T1DM group. The potent anti-inflammatory effects of *cafestol* were demonstrated by the dose-dependent reduction of renal IL-6, TNF-α, ICAM-1, and nuclear NF-κB. This suggests that *cafestol* affects the inflammatory environment, potentially through blocking NF-κB signalling, a crucial modulator of the expression of inflammatory genes [59]. This is consistent with studies that demonstrate that *cafestol* inhibits inflammatory mediators in various disease models, typically due to its antioxidant properties and alteration of inflammatory processes [21,60]. Further mechanistic analysis revealed that Nrf2 signalling, when Brusatol and *Cafestol* (10 mg/kg) were given together, the protective effects were removed in the T1DM group. Reversal was indicated by the sharp rise in renal levels of IL-6, TNF-α, ICAM-1, and nuclear NF-κB compared to the levels of untreated T1DM.

This clearly shows that the anti-inflammatory activity of *Cafestol* occurs due to its activation of the Nrf2 pathway. Nrf2 is a key transcription factor that helps the body fight against oxidative stress. *Cafestol* can activate it, and this leads to its signalling pathways that can help to induce protection from diabetic nephropathy [61,62]. Studies have shown the complex crosstalk between Nrf2 and NF-κB pathways. Further, Nrf2 activation can inhibit NF-κB activity and downregulate the biosynthesis of pro-inflammatory cytokines [63]. In line with the present findings, evidence for such an interaction pertaining to the therapeutic beneficial action of Cafestol is strong.

The administration of *Cafestol* (10 mg/kg) in control rats reduced serum AGEs and sRAGEs, as well as kidney MDA, while increasing antioxidant indicators such as GSH, HO-1, and SOD, thus illustrating its antioxidative and anti-glycation properties. In T1DM rats, elevated serum AGEs, sRAGEs, and renal MDA, together with decreased antioxidant indicators, signified substantial oxidative stress and lower antioxidant capability typical of diabetes. *Cafestol* administration dose-dependently ameliorated these anomalies in T1DM mice, reducing oxidative damage and reinstating antioxidant enzyme levels, aligning with its function in alleviating diabetes-induced oxidative stress and glycation [21,64]. The elimination of these protective benefits with the co-administration of Brusatol, an HO-1 inhibitor, substantiates the essential function of HO-1 induction in *Cafestol’s* mode of action [64]. These results correspond with recent research emphasizing *Cafestol’s* capacity to augment antioxidant defenses, diminish glycation end products, and facilitate glucose metabolism regulation in diabetic models, thereby reinforcing its therapeutic potential for managing oxidative and glycation stress associated with diabetes [45,47,65].

*Cafestol* diminishes Keap1, thereby augmenting Nrf2 signaling and antioxidant defense in both normal and diabetic rats; Brusatol inhibits this effect, validating *Cafestol’s* Nrf2-dependent protective mechanism. This effect aligns with recent studies demonstrating *Cafestol’s* potential to promote Nrf2 nuclear translocation and subsequent antioxidant gene expression, therefore mitigating oxidative stress and inflammation in renal and cardiac tissues [23,64]. The regulation of the Keap1/Nrf2 pathway is an established therapeutic target for diabetic complications, as Nrf2 activation alleviates oxidative damage associated with diabetic nephropathy [66,67].

When compared to the T1DM group, T1DM + *Cafestol* (10 mg/kg) + Brusatol rats showed no significant change in these markers in the kidney. Comparing these two models reinforces the idea that the Nrf2 pathway is a major mediator of *Cafestol’s* anti-inflammatory effects since blocking the Nrf2 pathway abolishes the protective effects and results in an inflammatory pattern comparable to that seen under diabetic conditions, where Nrf2 activation is lost or impaired. As per the above results, the Nrf2-dependent anti-inflammatory mechanism of *cafestol* has potential in combating DN. The current study has some limitations. First, mechanistic conclusions have been drawn mainly from a streptozotocin-induced T1DM rat model and the use of the pharmacological inhibitor, Brusatol; thus, while these data support the hypothesized role of the renal Keap1-Nrf2 axis in mediating the effects of *cafestol* on renal Nrf2 activation, experimental validation at the cellular level via gene-specific methods (i.e., siRNA or CRISPR/Cas9) to confirm Nrf2 activation induced by *cafestol* in renal cells subjected to high-glucose conditions was not performed. Second, although changes in Nrf2 levels within both the cytoplasmic and nuclear compartments of the kidney were measured in fractionated extracts from the kidney (and shown to correlate with corresponding changes in expression of Keap1 and antioxidant markers), the level of nuclear Nrf2 was not validated by either Western blot or immunofluorescent analysis, both of which would have provided stronger, more semi-quantitative support for Nrf2 redistribution at the subcellular level. Third, a limited panel of antioxidant mediators downstream of Nrf2 (HO-1, SOD, and GSH) were evaluated, as opposed to performing a more extensive analysis of the classical Nrf2 targets at either the mRNA or protein level. Lastly, while histological evaluations were performed, the majority of these analyses were qualitative in nature and morphometric determinations of several renal brunches (e.g., glomerular volume, mesangial expansion, and tubular injury scores) were not completed. Fifthly, even though the initial doses selected for *cafestol* (5 mg/kg and 10 mg/kg) were based on their preliminary dose-response characteristics as well as previous empirical support, there were no plasma or renal tissue concentrations of *cafestol* assessed; therefore, we cannot definitively prove that these dosing levels have direct physiological relevance or translational equivalence. Also, the design of this study was for an endpoint design over an 8 weeks period, which was relevant to the development of diabetic nephropathy in the present model system; however, there was no time-point analysis so it is unknown what temporal relationship Nrf2 activation has to later changes in renal function, oxidative stress, and inflammation. Future studies should include renal cell-type specific genetic validation, broader profiling of Nrf2 regulated genes, quantitative analysis of renal morphology, pharmacokinetic evaluation and time course analysis of how long it takes for *cafestol* to exert its renoprotective effects (at intervals of 2 weeks up until 2 weeks and 4 weeks) to properly establish the molecular and temporal dynamics of *cafestol* mediated renoprotection

## Conclusions

The present findings reveal that streptozotocin-induced T1DM is associated with severe metabolic, lipid, renal, and inflammatory disorders, which are consistent with previous diabetic models. Although cafestol did not improve hyperglycemia, dyslipidemia, or other systemic metabolic disorders related to T1DM at the doses tested, it had significant renoprotective and anti-inflammatory actions. Cafestol significantly decreased the parameters of diabetic nephropathy, such as albuminuria, urinary injury biomarkers (KIM-1, NGAL, nephrin), and pro-inflammatory mediators (IL-6, TNF-α, ICAM-1, and NF-κB activation) in a dose-dependent fashion. These protective actions were completely lost by the concomitant administration of brusatol, an Nrf2 inhibitor, indicating that the beneficial renal and anti-inflammatory effects of cafestol are largely exerted through the activation of the Nrf2 signaling pathway. Collectively, the data indicates that while cafestol does not restore systemic metabolic dysfunction in T1DM, it nonetheless exerts significant protection against renal oxidative and inflammatory insult. The outcome underlines the therapeutic potential of cafestol as a Nrf2-dependent renoprotective agent in diabetic nephropathy and justifies further study on optimal dosing, molecular mechanisms, and applicability in broader diabetic contexts.

## Supporting information

S1 File Orginal data- cafestol. (PDF)

## References

[1] Tuttle KR, Bakris GL, Bilous RW, Chiang JL, de Boer IH, Goldstein-Fuchs J. Diabetic kidney disease: A report from an ADA Consensus Conference. Diabetes Care. 2022;45(12):3073–89. doi: 10.2337/dc14-1296 PMC4170131 25249672

[2] Wang Y, Jin M, Cheng CK, Li Q. Tubular injury in diabetic kidney disease: Molecular mechanisms and potential therapeutic perspectives. Front Endocrinol (Lausanne). 2023;14:1238927. doi: 10.3389/fendo.2023.1238927 37600689 PMC10433744

[3] Kawanami D, Matoba K, Utsunomiya K. Dyslipidemia in diabetic nephropathy. Ren Replace Ther. 2016;2(1). doi: 10.1186/s41100-016-0028-0

[4] Mahmoodnia L, Aghadavod E, Beigrezaei S, Rafieian-Kopaei M. An update on diabetic kidney disease, oxidative stress and antioxidant agents. J Renal Inj Prev. 2017;6(2):153–7. doi: 10.15171/jrip.2017.30 28497094 PMC5423285

[5] Rico-Fontalvo J, Aroca G, Cabrales J, Daza-Arnedo R, Yánez-Rodríguez T, Martínez-Ávila MC, et al. Molecular mechanisms of diabetic kidney disease. Int J Mol Sci. 2022;23(15):8668. doi: 10.3390/ijms23158668 35955802 PMC9369345

[6] Pichler R, Afkarian M, Dieter BP, Tuttle KR. Immunity and inflammation in diabetic kidney disease: Translating mechanisms to biomarkers and treatment targets. Am J Physiol Renal Physiol. 2017;312(4):F716–31. doi: 10.1152/ajprenal.00314.2016 27558558 PMC6109808

[7] Nithya R, Subramanian S. Sinapic acid, a naturally occurring carboxylic acid derivative ameliorates hyperglycemia in high fat diet-low dose stz induced experimental diabetic rats. Int J Sci Eng Tech Res. 2015;4:5746–50.

[8] Klein G, Kim J, Himmeldirk K, Cao Y, Chen X. Antidiabetes and anti-obesity activity of Lagerstroemia speciosa. Evid Based Complement Alternat Med. 2007;4(4):401–7. doi: 10.1093/ecam/nem013 18227906 PMC2176148

[9] Alicic RZ, Johnson EJ, Tuttle KR. Inflammatory mechanisms as new biomarkers and therapeutic targets for diabetic kidney disease. Adv Chronic Kidney Dis. 2018;25(2):181–91. doi: 10.1053/j.ackd.2017.12.002 29580582

[10] Lu Z, Zhong Y, Liu W, Xiang L, Deng Y. The efficacy and mechanism of chinese herbal medicine on diabetic kidney disease. J Diabetes Res. 2019;2019:2697672. doi: 10.1155/2019/2697672 31534972 PMC6732610

[11] Sun W, Liu X, Zhang H, Song Y, Li T, Liu X, et al. Epigallocatechin gallate upregulates NRF2 to prevent diabetic nephropathy via disabling KEAP1. Free Radic Biol Med. 2017;108:840–57. doi: 10.1016/j.freeradbiomed.2017.04.365 28457936

[12] Dong W, Jia Y, Liu X, Zhang H, Li T, Huang W, et al. Sodium butyrate activates NRF2 to ameliorate diabetic nephropathy possibly via inhibition of HDAC. J Endocrinol. 2017;232(1):71–83. doi: 10.1530/JOE-16-0322 27799462

[13] Chen Y-J, Kong L, Tang Z-Z, Zhang Y-M, Liu Y, Wang T-Y, et al. Hesperetin ameliorates diabetic nephropathy in rats by activating Nrf2/ARE/glyoxalase 1 pathway. Biomed Pharmacother. 2019;111:1166–75. doi: 10.1016/j.biopha.2019.01.030 30841430

[14] Xing L, Guo H, Meng S, Zhu B, Fang J, Huang J, et al. Klotho ameliorates diabetic nephropathy by activating Nrf2 signaling pathway in podocytes. Biochem Biophys Res Commun. 2021;534:450–6. doi: 10.1016/j.bbrc.2020.11.061 33256980

[15] Kidokoro K, Cherney DZI, Bozovic A, Kashihara N. The role of SGLT2 inhibitors in the treatment of diabetic kidney disease. Curr Diab Rep. 2020;20(9):37. doi: 10.3390/pharmaceutics1507199532638126

[16] Heerspink HJL, Stefánsson BV, Correa-Rotter R, Chertow GM, Greene T, Hou F-F, et al. Dapagliflozin in patients with chronic kidney disease. N Engl J Med. 2020;383(15):1436–46. doi: 10.1056/NEJMoa2024816 32970396

[17] Zhang B, Zhou L, Chen K, Fang X, Li Q, Gao Z, et al. Investigation on phenomics of traditional chinese medicine from the diabetes. Phenomics. 2024;4(3):257–68. doi: 10.1007/s43657-023-00146-6 39398423 PMC11467137

[18] Zhou T-Y, Tian N, Li L, Yu R. Iridoids modulate inflammation in diabetic kidney disease: A review. J Integr Med. 2024;22(3):210–22. doi: 10.1016/j.joim.2024.03.010 38631983

[19] Akash MSH, Rehman K, Chen S. Effects of coffee on type 2 diabetes mellitus. Nutrition. 2014;30(7–8):755–63. doi: 10.1016/j.nut.2013.11.020 24984989

[20] Ding M, Bhupathiraju SN, Satija A, van Dam RM, Hu FB. Long-term coffee consumption and risk of cardiovascular disease: A systematic review and a dose-response meta-analysis of prospective cohort studies. Circulation. 2014;129(6):643–59. doi: 10.1161/CIRCULATIONAHA.113.005925 24201300 PMC3945962

[21] Mellbye FD, Hermansen K, Jeppesen PB, Gregersen S. Acute effects of the coffee diterpene cafestol on glucose metabolism in non-diabetic subjects with abdominal obesity. The Review of Diabetic Studies. 2023;19:34. doi: 10.1900/RDS.2023.19.34

[22] Ren X, Wang M, Chen J, Cao R, Sun Y, Jin H. Protective effects of cafestol and kahweol against oxidative stress: A review. Food Research International. 2019;116:322–32. doi: 10.3390/ijms20174238

[23] Al-Kenany E, Auda G, El-Desoky K, El-Naggar S. Cafestol exerts protective effects against doxorubicin-induced cardiac toxicity via Nrf2/HO-1 and NF-κB pathways. Biomed Pharmacother. 2023;157:114011. doi: 10.3389/fphar.2023.120678236410123

[24] Liu J-C, Chen P-Y, Hao W-R, Liu Y-C, Lyu P-C, Hong H-J. Cafestol inhibits high-glucose-induced cardiac fibrosis in cardiac fibroblasts and type 1-like diabetic rats. Evid Based Complement Alternat Med. 2020;2020:4503747. doi: 10.1155/2020/4503747 33488743 PMC7790572

[25] Mellbye FB, Jeppesen PB, Shokouh P, Laustsen C, Hermansen K, Gregersen S. Cafestol, a bioactive substance in coffee, has antidiabetic properties in KKAy mice. J Nat Prod. 2017;80(8):2353–9. doi: 10.1021/acs.jnatprod.7b00395 28763212

[26] Huxley R, Lee CMY, Barzi F, Timmermeister L, Czernichow S, Perkovic V, et al. Coffee, decaffeinated coffee, and tea consumption in relation to incident type 2 diabetes mellitus: A systematic review with meta-analysis. Arch Intern Med. 2009;169(22):2053–63. doi: 10.1001/archinternmed.2009.439 20008687

[27] Kim JY, Jung KS, Jeong HG. Suppressive effects of the kahweol and cafestol on cyclooxygenase-2 expression in macrophages. FEBS Lett. 2004;569(1–3):321–6. doi: 10.1016/j.febslet.2004.05.070 15225655

[28] Shen C-P, Luo J-G, Yang M-H, Kong L-Y. Cafestol-type diterpenoids from the twigs of tricalysia fruticosa with potential anti-inflammatory activity. J Nat Prod. 2015;78(6):1322–9. doi: 10.1021/acs.jnatprod.5b00165 26052978

[29] Hao W-R, Sung L-C, Chen C-C, Chen P-Y, Cheng T-H, Chao H-H, et al. Cafestol inhibits cyclic-strain-induced interleukin-8, intercellular adhesion molecule-1, and monocyte chemoattractant protein-1 production in vascular endothelial cells. Oxid Med Cell Longev. 2018;2018:7861518. doi: 10.1155/2018/7861518 29854096 PMC5952558

[30] AlMousa LA, AlFaris NA, Alshammari GM, Alsayadi MM, ALTamimi JZ, Alagal RI, et al. Rumex nervosus could alleviate streptozotocin-induced diabetic nephropathy in rats by activating Nrf2 signaling. Sci Prog. 2022;105(2):368504221102751. doi: 10.1177/00368504221102751 35619568 PMC10358522

[31] ALTamimi JZ, AlFaris NA, Alshammari GM, Alagal RI, Aljabryn DH, Yahya MA. Esculeoside a decreases diabetic cardiomyopathy in streptozotocin-treated rats by attenuating oxidative stress, inflammation, fibrosis, and apoptosis: Impressive Role of Nrf2. Medicina (Kaunas). 2023;59(10):1830. doi: 10.3390/medicina59101830 37893548 PMC10608477

[32] Pei D, Tian S, Bao Y, Zhang J, Xu D, Piao M. Protective effect of salidroside on streptozotocin-induced diabetic nephropathy by inhibiting oxidative stress and inflammation in rats via the Akt/GSK-3β signalling pathway. Pharm Biol. 2022;60(1):1732–8. doi: 10.1080/13880209.2022.2116055 36086879 PMC9467606

[33] Yahya MA, Alshammari GM, Osman MA, Al-Harbi LN, Yagoub AEA, AlSedairy SA. Isoliquiritigenin attenuates high-fat diet-induced intestinal damage by suppressing inflammation and oxidative stress and through activating Nrf2. Journal of Functional Foods. 2022;92:105058. doi: 10.1016/j.jff.2022.105058

[34] Hao W-R, Cheng C-Y, Chen H-Y, Chen J-J, Cheng T-H, Liu J-C. The Association between Cafestol and Cardiovascular Diseases: A comprehensive review. Medicina (Kaunas). 2024;60(6):867. doi: 10.3390/medicina60060867 38929484 PMC11205330

[35] Mellbye FD, Nguyen MD, Hermansen K, Jeppesen PB, Al-Mashhadi ZK, Ringgaard S, et al. Effects of 12-Week supplementation with coffee diterpene cafestol in healthy subjects with increased waist circumference: A randomized, placebo-controlled trial. Nutrients. 2024;16(19):3232. doi: 10.3390/nu16193232 39408200 PMC11478357

[36] Mellbye FD, Hermansen K, Jeppesen PB, Gregersen S. Cafestol and kahweol acutely reduce glucose levels in subjects with type 2 diabetes. The Review of Diabetic Studies. 2025;21(1). doi: 10.1900/4j5q0416

[37] Al-Malki AL, El Rabey HA. The antidiabetic effect of low doses of Moringa oleifera Lam. seeds on streptozotocin induced diabetes and diabetic nephropathy in male rats. BioMed Research International. 2015;2015(1):381040. 25629046 10.1155/2015/381040 PMC4299558

[38] Si Q, Guo J, Yang X, Guo Y, Wu L, Xie D, et al. Systematic assessment of streptozotocin-induced diabetic metabolic alterations in rats using metabolomics. Front Endocrinol (Lausanne). 2023;14:1107162. doi: 10.3389/fendo.2023.1107162 36761194 PMC9902650

[39] Mediani A, Abas F, Maulidiani M, Khatib A, Tan CP, Ismail IS, et al. Metabolic and biochemical changes in streptozotocin induced obese-diabetic rats treated with Phyllanthus niruri extract. J Pharm Biomed Anal. 2016;128:302–12. doi: 10.1016/j.jpba.2016.06.003 27318080

[40] Fernández-Ochoa Á, Cázares-Camacho R, Borrás-Linares I, Domínguez-Avila JA, Segura-Carretero A, González-Aguilar GA. Evaluation of metabolic changes in liver and serum of streptozotocin-induced diabetic rats after Mango diet supplementation. Journal of Functional Foods. 2020;64:103695. doi: 10.1016/j.jff.2019.103695

[41] Ugarte M, Brown M, Hollywood KA, Cooper GJ, Bishop PN, Dunn WB. Metabolomic analysis of rat serum in streptozotocin-induced diabetes and after treatment with oral triethylenetetramine (TETA). Genome Med. 2012;4(4):35. doi: 10.1186/gm334 22546713 PMC3446263

[42] Schein PS, Alberti KG, Williamson DH. Effects of streptozotocin on carbohydrate and lipid metabolism in the rat. Endocrinology. 1971;89(3):827–34. doi: 10.1210/endo-89-3-827 4327777

[43] Sheng Y, Zheng S, Ma T, Zhang C, Ou X, He X, et al. Mulberry leaf alleviates streptozotocin-induced diabetic rats by attenuating NEFA signaling and modulating intestinal microflora. Sci Rep. 2017;7(1):12041. doi: 10.1038/s41598-017-12245-2 28935866 PMC5608946

[44] Sudnikovich EJ, Maksimchik YZ, Zabrodskaya SV, Kubyshin VL, Lapshina EA, Bryszewska M, et al. Melatonin attenuates metabolic disorders due to streptozotocin-induced diabetes in rats. Eur J Pharmacol. 2007;569(3):180–7. doi: 10.1016/j.ejphar.2007.05.018 17597602

[45] Gomes DS, Romanelli MA, Gomes SPS, Brand ALM, da Silva RMV, Oliveira SSC, et al. Pharmacological potential of cafestol, a bioactive substance in coffee, in preventing ischemia-reperfusion-induced acute kidney injury. ACS Omega. 2025;10(22):22825–36. doi: 10.1021/acsomega.4c11728 40521450 PMC12163779

[46] Jiang T, Huang Z, Lin Y, Zhang Z, Fang D, Zhang DD. The protective role of Nrf2 in streptozotocin-induced diabetic nephropathy. Diabetes. 2010;59(4):850–60. doi: 10.2337/db09-1342 20103708 PMC2844833

[47] Bondi CD, Hartman HL, Tan RJ. NRF2 in kidney physiology and disease. Physiol Rep. 2024;12(5):e15961. doi: 10.14814/phy2.15961 38418382 PMC10901725

[48] Guerrero-Hue M, Rayego-Mateos S, Vázquez-Carballo C, Palomino-Antolín A, García-Caballero C, Opazo-Rios L, et al. Protective Role of Nrf2 in renal disease. Antioxidants (Basel). 2020;10(1):39. doi: 10.3390/antiox10010039 33396350 PMC7824104

[49] Hashemi M, Zandieh MA, Ziaolhagh S, Mojtabavi S, Sadi FH, Koohpar ZK, et al. Nrf2 signaling in diabetic nephropathy, cardiomyopathy and neuropathy: Therapeutic targeting, challenges and future prospective. Biochim Biophys Acta Mol Basis Dis. 2023;1869(5):166714. doi: 10.1016/j.bbadis.2023.166714 37028606

[50] Cui W, Min X, Xu X, Du B, Luo P. Role of nuclear factor erythroid 2-related factor 2 in diabetic nephropathy. J Diabetes Res. 2017;2017:3797802. doi: 10.1155/2017/3797802 28512642 PMC5420438

[51] Foresto-Neto O, Albino AH, Arias SCA, Faustino VD, Zambom FFF, Cenedeze MA, et al. NF-κB system is chronically activated and promotes glomerular injury in experimental type 1 diabetic kidney disease. Front Physiol. 2020;11:84. doi: 10.3389/fphys.2020.00084 32116790 PMC7026681

[52] Dodson M, Shakya A, Anandhan A, Chen J, Garcia JGN, Zhang DD. NRF2 and diabetes: The good, the bad, and the complex. Diabetes. 2022;71(12):2463–76. doi: 10.2337/db22-0623 36409792 PMC9750950

[53] Wu J, Sun X, Jiang Z, Jiang J, Xu L, Tian A, et al. Protective role of NRF2 in macrovascular complications of diabetes. J Cell Mol Med. 2020;24(16):8903–17. doi: 10.1111/jcmm.15583 32628815 PMC7417734

[54] Tanase DM, Gosav EM, Anton MI, Floria M, Seritean Isac PN, Hurjui LL, et al. Oxidative Stress and NRF2/KEAP1/ARE Pathway in Diabetic Kidney Disease (DKD): New perspectives. Biomolecules. 2022;12(9):1227. doi: 10.3390/biom12091227 36139066 PMC9496369

[55] Jin Q, Liu T, Qiao Y, Liu D, Yang L, Mao H, et al. Oxidative stress and inflammation in diabetic nephropathy: Role of polyphenols. Front Immunol. 2023;14:1185317. doi: 10.3389/fimmu.2023.1185317 37545494 PMC10401049

[56] Yi M, Toribio AJ, Salem YM, Alexander M, Ferrey A, Swentek L, et al. Nrf2 signaling pathway as a key to treatment for diabetic dyslipidemia and atherosclerosis. Int J Mol Sci. 2024;25(11):5831. doi: 10.3390/ijms25115831 38892018 PMC11172493

[57] Zhang L, Xu F, Hou L. IL-6 and diabetic kidney disease. Front Immunol. 2024;15:1465625. doi: 10.3389/fimmu.2024.1465625 39749325 PMC11693507

[58] Donate-Correa J, Ferri CM, Sánchez-Quintana F, Pérez-Castro A, González-Luis A, Martín-Núñez E, et al. Inflammatory cytokines in diabetic kidney disease: Pathophysiologic and therapeutic implications. Front Med (Lausanne). 2021;7:628289. doi: 10.3389/fmed.2020.628289 33553221 PMC7862763

[59] Mohamed AI, Erukainure OL, Salau VF, Islam MS. Impact of coffee and its bioactive compounds on the risks of type 2 diabetes and its complications: A comprehensive review. Diabetes Metab Syndr. 2024;18(7):103075. doi: 10.1016/j.dsx.2024.103075 39067326

[60] Surma S, Sahebkar A, Banach M. Coffee or tea: Anti-inflammatory properties in the context of atherosclerotic cardiovascular disease prevention. Pharmacol Res. 2023;187:106596. doi: 10.1016/j.phrs.2022.106596 36473629

[61] Gao W, Guo L, Yang Y, Wang Y, Xia S, Gong H, et al. Dissecting the crosstalk between Nrf2 and NF-κB response pathways in drug-induced toxicity. Front Cell Dev Biol. 2022;9:809952. doi: 10.3389/fcell.2021.809952 35186957 PMC8847224

[62] Althobaiti F, Taher ES, Ahmed Alkeridis L, Ibrahim AM, El-Shafai N, A Al-Shuraym L, et al. Exploring the NRF2/HO-1 and NF-κB Pathways: Spirulina nanoparticles as a novel approach to combat diabetic nephropathy. ACS Omega. 2024;9(22):23949–62. doi: 10.1021/acsomega.4c02285 38854532 PMC11154939

[63] Ganesh Yerra V, Negi G, Sharma SS, Kumar A. Potential therapeutic effects of the simultaneous targeting of the Nrf2 and NF-κB pathways in diabetic neuropathy. Redox Biol. 2013;1(1):394–7. doi: 10.1016/j.redox.2013.07.005 24024177 PMC3757712

[64] Mellbye FB, Jeppesen PB, Hermansen K, Gregersen S. Cafestol, a bioactive substance in coffee, stimulates insulin secretion and increases glucose uptake in muscle cells: Studies in Vitro. J Nat Prod. 2015;78(10):2447–51. doi: 10.1021/acs.jnatprod.5b00481 26465380

[65] Chen P-M, Gregersen H, Zhao J-B. Advanced glycation end-product expression is upregulated in the gastrointestinal tract of type 2 diabetic rats. World J Diabetes. 2015;6(4):662–72. doi: 10.4239/wjd.v6.i4.662 25987965 PMC4434088

[66] Hao W-R, Sung L-C, Chen C-C, Hong H-J, Liu J-C, Chen J-J. Cafestol activates nuclear factor erythroid-2 related factor 2 and inhibits urotensin ii-induced cardiomyocyte hypertrophy. Am J Chin Med. 2019;47(2):337–50. doi: 10.1142/S0192415X19500162 30871360

[67] Lou Y, Kong M, Li L, Hu Y, Zhai W, Qi X, et al. Inhibition of the Keap1/Nrf2 signaling pathway significantly promotes the progression of type 1 diabetes mellitus. Oxid Med Cell Longev. 2021;2021:7866720. doi: 10.1155/2021/7866720 33628382 PMC7884168

