# Supplementary material for: Cafestol ameliorates diabetic nephropathy via Keap1-Nrf2 axis activation: A novel renoprotective mechanism independent of glycemic control
Source: PLoS One. 2026 May 14;21(5):e0349192. doi: 10.1371/journal.pone.0349192 (PMC13175328; doi:10.1371/journal.pone.0349192)
Supplement: S1 File — (PDF) [file pone.0349192.s001.pdf]

	Control	Control + Cafestol (10 mg/kg)	T1DM
	12.3	9.6	23.4
	11.4	13.2	20.9
	12.4	11.7	21.4
	10.87	10.9	24.5
	11.5	13.1	22.7
24-h urine volume	13.2	11.4	23.1
	12.5	10.6	23.8
	10.9	12.4	22.4

	Control	Control + Cafestol (10 mg/kg)	T1DM
	25.6	20	310
	28.7	32	329
Urine Albumin	22.4	26	372
	31.3	29	287
	28.5	24	318
	26.5	21	328
	28	25	338
	23	29	298

	Control	Control + Cafestol (10 mg/kg)	T1DM
	1145	894	221
Urine creatinine	1084	1632	198
	1343	1423	198
	965	1584	136
	1093	1392	202
	1432	1193	174
	1173	1094	183
	1284	1421	214

	Control	Control + Cafestol (10 mg/kg)	T1DM
	109	100	617
	98	128	549
	124	107	734
	132	111	669
	83	79.4	593
Urine -KIM1	115	89.3	400
	92.4	131	689
	126	85	593

	Control	Control + Cafestol (10 mg/kg)	T1DM
	45.6	26.7	189
	37.8	44	217
	41.4	33	200
	55.6	48	198
	31.3	36	178
Urine NGAL	49.2	58	194
	33.5	51	154

	61.3	65	176
	129	109	443
	154	115	378
	139	158	510
	131	163	444
	127	154	379
Urine nephrine	141	133	410
	139	120	400
	133	140	423

	Control	Control + Cafestol (10 mg/kg)	T1DM
	15.6	13.8	44.3
IL-6	18.5	14.5	49.5
	17.4	19.3	55
	13.2	18.3	51
	14.6	16.4	47
	16.8	15.3	43
	19.3	17.4	48
	14.3	14	46.8

	Control	Control + Cafestol (10 mg/kg)	T1DM
	6.5	3.6	26.7
	4.7	4.9	30.44
	8	7.6	27.6
TNF	5.4	7.1	33.2
	5.9	6.8	36.5
	6.3	6.5	29
	8	5	26.8
	6.3	9	38.5

	Control	Control + Cafestol (10 mg/kg)	T1DM
	534	334	1034
	344	556	1145
ICAM	419	603	1432
	445	209	983
	378	448	1000
	505	412	938
	436	299	1045
	493	377	1138

	Control	Control + Cafestol (10 mg/kg)	T1DM
	332	292	634
	209	219	509
	219	209	559
Nuclear NF-kb	283	226	516
	189	219	627
	209	287	592
	252	300	536

	267	217	548
	Control	Control + Cafestol (10 mg/kg)	T1DM
	13.4	6.7	46.7
	11.3	7.6	66.7
Serum AGEs	14.5	8.6	54.6
	16.5	8.9	55.4
	10.4	5.6	47.8
	13.6	8.7	48.9
	10.9	7.5	57.6
	9.8	7.1	51
	Control	Control + Cafestol (10 mg/kg)	T1DM
	1.3	0.73	14.5
	2.34	1.23	17.4
	2.45	1.1	13.4
Serum sRAGEs	3.45	0.81	12.8
	2.56	0.29	14.3
	1.89	1.42	10.9
	2.45	0.58	12.8
	2.743	0.74	11.9
	28.9	41.4	12.3
	30.3	38.9	11.3
	26.7	33.5	10.9
	25.6	37.6	8.7
HO-1	28.9	42.3	13.4
	25.4	36.7	9.5
	30.1	46.5	10.9
	33.1	41.3	12.4
	16.4	27.6	5.7
	14.5	25.6	4.9
SOD	15.6	29.4	6.4
	17.5	36.5	3.98
	15.4	33.2	4.4
	19.3	27.5	5.9
	14.5	28.9	6.3
	67.5	78.3	24.5
GSH	55.7	71.2	28.5
	58.7	66.4	28.5
	49.5	69.3	34.3
	60.2	66.8	22.3
	45.8	68.8	25.6
	52.5	57.4	26.8
	59.4	61.4	30.3

	Control	Control + Cafestol (10 mg/kg)	T1DM
	0.43	0.28	1.34
MDA	0.57	0.32	1.19
	0.47	0.42	1.46
	0.38	0.19	1.39
	0.55	0.38	1.29
	0.48	0.23	1.43
	0.53	0.399	1.17
	0.53	0.26	1.26

	Control	Control + Cafestol (10 mg/kg)	T1DM
	1.45	0.83	3.22
	1.32	0.92	3.12
keap1 mRNA	1.65	0.55	3.82
	1.33	0.75	2.5
	1.58	0.73	2.62
	1.29	0.82	2.98
	1.19	0.64	3.29
	1.45	0.68	3

	Control	Control + Cafestol (10 mg/kg)	T1DM
	0.834	0.75	0.74
	0.94	0.64	0.84
	0.78	0.99	0.93
Nrf2 mRNA	0.58	1	0.61
	0.88	0.65	0.66
	0.95	0.77	0.83
	0.74	0.69	0.59
	0.79	0.88	0.85

	Control	Control + Cafestol (10 mg/kg)	T1DM
Nrf2-cytoplasmic	554	833	224
	545	837	187
	456	923	209
	509	767	267
	604	793	217
	532	863	319
	598	814	167
	478	824	209

	Control	Control + Cafestol (10 mg/kg)	T1DM
	128	186	44.3
	135	173	41.3
Nrf2 nuclear	128	168	64
	115	192	55
	138	188	41
	118.3	172	53
	139	167	41
	127	174	49

	454.05	443.60	304.10
	474.70	471.40	318.40
	495.35	499.20	332.70
	516.00	527.00	347.00
Final BWt	516.00	527.00	347.00
	536.65	554.80	361.30
	557.30	582.60	375.60
	577.95	610.40	389.90

	222.85	218.14	318.85
	232.32	226.67	331.75
	241.79	235.2	344.65
	251.26	243.73	357.55
Food intake	260.74	252.27	370.45
	270.21	260.8	383.35
	279.68	269.33	396.25
	289.15	277.86	409.15
	85.36	84.8	300.78
	88.54	89.08	312.9
	91.72	93.37	325.02
GLucose	94.91	97.66	337.14
	98.09	101.94	349.26
	101.28	106.23	361.38
	104.46	110.52	373.5
	107.64	114.8	385.62
		99.8	343.2
	7.79812615	10.49956326	29.68781568
	3.74	3.29	7.3
	3.9	3.52	7.67
	4.06	3.75	8.04
	4.22	3.98	8.42
	4.38	4.22	8.78
Insulin	4.54	4.45	9.16
	4.7	4.68	9.53
	4.86	4.91	9.9
	4.47	4.1	6.68
HbA1C	4.81	4.36	6.94
	5.14	4.62	7.21
	5.48	4.88	7.48
	5.12	5.12	7.72
	5.46	5.38	7.99
	5.79	5.64	8.26
	6.13	5.9	8.52

	67.55	77.29	197.91
	71.52	80.4	208.36
	75.48	83.5	218.8
Serum TGs	79.44	86.61	229.25
	83.36	90.39	239.75
	87.32	93.5	250.2
	91.28	96.6	260.64
	95.25	99.71	271.09
	80.38	75.38	217.32
	84.67	79.13	228.55
	88.96	82.88	239.77
	93.25	86.63	251
	97.55	89	262.4
Serum CHOL	101.84	92.75	273.62
	106.13	96.5	284.85
	110.42	100.25	296.07
	6.41	33.84	103.78
	38.78	35.51	109.7
	41.15	37.19	115.62
LDL-c	43.52	38.86	121.54
	45.89	40.54	127.46
	48.25	42.21	133.38
	50.62	43.89	139.3
	52.99	45.56	145.22
	25.71	31.6	13.69
	27.3	33.48	14.26
	28.89	35.36	14.83
	30.48	37.24	15.4
HDL	32.12	37.96	15.99
	33.71	39.84	16.56
	35.3	41.72	17.13
	36.89	43.6	17.7

T1DM + Cafestol (5 mg/kg)	T1DM + Cafestol (10 mg/kg)	T1DM + Cafestol (10 mg/kg) + Brusatol
18.7	13.2	23.5
16.7	11.4	22.4
17.8	10.9	20.9
15.4	12.5	24.3
16.5	11.7	22.6
18.4	12.6	21.5
16.2	11.9	23.8
15.9	12.9	21.7

T1DM + Cafestol (5 mg/kg)	T1DM + Cafestol (10 mg/kg)	T1DM + Cafestol (10 mg/kg) + Brusatol
194.3	77	346
185.6	71	300
188.5	81	314
176.4	64	284
193.4	72	219
200	83	334
175	81	341
192	74	308

T1DM + Cafestol (5 mg/kg)	T1DM + Cafestol (10 mg/kg)	T1DM + Cafestol (10 mg/kg) + Brusatol
843	1321	202
732	943	110
912	1173	105
658	1453	288
771	1322	135
604	1209	178
813	1093	100
649	1443	216

T1DM + Cafestol (5 mg/kg)	T1DM + Cafestol (10 mg/kg)	T1DM + Cafestol (10 mg/kg) + Brusatol
332	192	722
411	154	593
374	132	609
326	183	463
382	109	594
354	178	775
317	153	520
369	143	588

T1DM + Cafestol (5 mg/kg)	T1DM + Cafestol (10 mg/kg)	T1DM + Cafestol (10 mg/kg) + Brusatol
101	91	219
98.4	83.4	178
105	81.9	234
122	77.3	189
89	58.6	198
119	76	209
91	65	200

86	79	187

298	187	499
256	200	483
248	156	392
278	163	356
239	179	381
243	187	399
250	198	459
251	183	482

T1DM + Cafestol (5 mg/kg)	T1DM + Cafestol (10 mg/kg)	T1DM + Cafestol (10 mg/kg) + Brusatol
32.3	18.4	47.8
31.3	16.5	61.2
37.3	18	55
29.5	22	48
34	20	45
28	24	58
36	18.4	51
33	15.9	50

T1DM + Cafestol (5 mg/kg)	T1DM + Cafestol (10 mg/kg)	T1DM + Cafestol (10 mg/kg) + Brusatol
15.6	8.4	34.5
17.4	6.5	28.4
19.4	5.9	26.7
15.4	9.3	29.5
13.4	8.4	31
14	7.4	25.6
12.9	7	28.5
16.7	6.1	26

T1DM + Cafestol (5 mg/kg)	T1DM + Cafestol (10 mg/kg)	T1DM + Cafestol (10 mg/kg) + Brusatol
745	345	1420
803	319	1023
635	493	839
784	419	999
728	327	1146
802	504	1289
792	552	1029
815	438	1151

T1DM + Cafestol (5 mg/kg)	T1DM + Cafestol (10 mg/kg)	T1DM + Cafestol (10 mg/kg) + Brusatol
429	163	539
328	282	702
310	208	772
372	198	548
453	288	666
389	234	503
334	219	498

362	226	614
T1DM + Cafestol (5 mg/kg)	T1DM + Cafestol (10 mg/kg)	T1DM + Cafestol (10 mg/kg) + Brusatol
32.4	15.6	46.7
25.6	13.2	61.4
33.5	10.3	58.9
27.8	15.4	55.6
30.2	17.5	43.4
33.4	12.6	58.9
27.9	12.1	61.2
32.4	11.9	57.3
T1DM + Cafestol (5 mg/kg)	T1DM + Cafestol (10 mg/kg)	T1DM + Cafestol (10 mg/kg) + Brusatol
5.4	2.1	15.9
7.5	2.9	13.4
8.4	1.9	14.9
6.9	3.1	9.5
7.5	2.4	11.8
7.2	2.3	13.7
8.3	2.7	12.8
6.9	2.91	11.8
19.3	33.4	7.98
22.4	29.8	14.3
18.5	34.5	11.3
16.5	23.4	9.5
20.9	26.7	13.4
15.6	24.3	11.8
18.5	34.5	14.3
20.4	31.9	11
13.4	18.4	6.9
10.3	14.9	5.4
9.5	17.5	5.9
13.4	17.4	7.3
11.2	16.4	2.7
10.3	18.9	5.4
9.6	16.4	5.7
45.6	64.6	21.3
38.5	73.4	18.7
38.6	62.3	25.6
43.5	66.8	33.4
35.6	59.6	23.4
43.5	51.5	22
40.4	63.4	26.7
47.5	60.2	21.4

T1DM + Cafestol (5 mg/kg)	T1DM + Cafestol (10 mg/kg)	T1DM + Cafestol (10 mg/kg) + Brusatol
0.78	0.43	1.39
0.63	0.38	1.43
0.89	0.58	1.118
0.78	0.48	1.39
0.77	0.51	1.19
0.63	0.34	1.47
0.73	0.48	1.29
0.69	0.41	1.27

T1DM + Cafestol (5 mg/kg)	T1DM + Cafestol (10 mg/kg)	T1DM + Cafestol (10 mg/kg) + Brusatol
2.13	1.35	3.54
2.432	1.57	3.21
2	1.63	3.7
2.52	1.22	2.8
2.39	1.39	2.78
2.15	1.45	3.22
2.28	1.1	3.14
2.66	1.63	3.48

T1DM + Cafestol (5 mg/kg)	T1DM + Cafestol (10 mg/kg)	T1DM + Cafestol (10 mg/kg) + Brusatol
0.64	0.883	0.61
0.92	0.81	0.59
0.81	0.71	0.67
0.83	0.64	0.84
0.73	0.89	0.9
0.79	0.94	0.64
0.83	0.83	0.95
0.63	0.67	0.73

T1DM + Cafestol (5 mg/kg)	T1DM + Cafestol (10 mg/kg)	T1DM + Cafestol (10 mg/kg) + Brusatol
376	593	198
389	529	236
343	557	200
306	498	278
372	509	235
329	528	292
400	620	192
339	600	216

T1DM + Cafestol (5 mg/kg)	T1DM + Cafestol (10 mg/kg)	T1DM + Cafestol (10 mg/kg) + Brusatol
99	143	53
114	139	39
83	128	61
82	104	51
91	136	48
81	153	42
100	122	49
85	119	61

296.75	296.10	311.25
315.00	311.40	326.00
333.25	326.70	340.75
351.50	342.00	355.50
351.50	342.00	355.50
369.75	357.30	370.25
388.00	372.60	385.00
406.25	387.90	399.75

295.7	302.28	306.7
311.5	315.63	320.5
327.3	328.98	334.3
343.1	342.33	348.1
358.9	355.67	361.9
374.7	369.02	375.7
390.5	382.37	389.5
406.3	395.72	403.3

272.46	276.5	288.26
286.47	294.5	304.3
300.48	312.5	320.34
314.49	330.5	336.38
328.51	348.5	352.42
342.52	366.5	368.46
356.53	384.5	384.5
370.54	402.5	400.54
321.5	339.5	344.4
34.32201709	44.09081537	39.28981547

6.65	6.91	7.46
7.01	7.25	7.84
7.36	7.59	8.22
7.72	7.94	8.61
8.08	8.26	8.99
8.44	8.61	9.38
8.79	8.95	9.76
9.15	9.29	10.14

6.84	5.85	6.64
7.15	6.21	6.99
7.45	6.56	7.34
7.76	6.92	7.69
8.04	7.28	7.91
8.35	7.64	8.26
8.65	7.99	8.61
8.96	8.35	8.96

203.41	231.66	201.41
212.96	239.3	213.43
222.51	246.95	225.44
232.05	254.59	237.45
241.75	262.21	249.35
251.29	269.85	261.36
260.84	277.5	273.38
270.39	285.14	285.39
230.36	231.39	208.19
242.64	243.39	220
254.93	255.4	231.81
267.21	267.4	243.61
279.59	279.41	255.39
291.87	291.41	267.19
304.16	303.42	279
316.44	315.42	290.81
104.06	111.67	100.65
108.47	116.45	106.12
112.88	121.23	111.6
117.29	126.01	117.06
121.71	130.79	122.54
126.12	135.57	128
130.53	140.35	133.48
134.94	145.13	138.95
14	12.39	13.79
14.73	13.16	14.64
15.46	13.93	15.49
16.19	14.7	16.34
17.01	15.5	17.26
17.74	16.27	18.11
18.47	17.04	18.96
19.2	17.81	19.81
